# Assessment and enrolment process for liver transplantation: nursing management through quality indicators

**DOI:** 10.1590/S1679-45082018AO3975

**Published:** 2018-04-06

**Authors:** Fabíola Faustino de Machado Dias, Samira Scalso de Almeida, Marcio Dias de Almeida

**Affiliations:** 1 Hospital Israelita Albert Einstein, São Paulo, SP, Brazil

**Keywords:** Liver transplantation, Quality indicators, health care, Quality of health care, Disease management, Patient selection, Transplante de fígado, Indicadores de qualidade em assistência à saúde, Qualidade da assistência à saúde, Gerenciamento clínico, Seleção de pacientes

## Abstract

**Objective:**

To establish, measure and analyze quality indicators in the evaluation and enrolment process of patients in a liver transplant program.

**Methods:**

A quantitative and non-experimental research, with data collected from the database of a liver transplant program, from September 2012 to September 2014. Descriptive statistics were used to analyze the quality indicators identified.

**Results:**

We analyzed 1,311 visits in the transplant outpatient clinic, most males (66.7%), white (65.1%), mean age of 53 (±12.5) years, from the Southeastern Region (91.2%), and from the State of São Paulo (80.8%). The indicators measured were efficiency of patient admission process (54.8%), efficiency of evaluation of transplant indication (39.9%), efficacy of treatment of patients seen in the program (21.8%), and waiting time to inclusion in the waiting list, median of 84 days (36-142).

**Conclusion:**

The quality indicators analyzed in this study enabled a quantitative view of the process, facilitating management of results and benchmarking with other transplant centers. Continuous monitoring can optimize resource allocation and planning of services in patient's admission process.

## RESUMO

**Objetivo:**

Estabelecer, mensurar e analisar indicadores de qualidade no processo de avaliação e admissão dos pacientes em um programa de transplante hepático.

**Métodos:**

Pesquisa de abordagem quantitativa não experimental, com dados coletados a partir do banco de dados da gestão de um programa de transplante hepático entre setembro de 2012 e setembro de 2014. Estatística descritiva foi utilizada para analisar os indicadores de qualidade identificados.

**Resultados:**

Foram analisados 1.311 atendimentos no ambulatório de transplante, a maioria do sexo masculino (66,7%), branca (65,1%), com média de idade de 53 (±12,5) anos, da Região Sudeste (91,2%) e do Estado de São Paulo (80,8%). Os indicadores mensurados foram eficiência do processo de admissão dos pacientes (54,8%), eficiência da avaliação de indicação ao transplante (39,9%), eficácia do tratamento dos pacientes atendidos no programa (21,8%), e tempo de inclusão na lista de espera, com mediana em 84 dias (36-142).

**Conclusão:**

Os indicadores de qualidade analisados neste estudo possibilitaram uma visão quantitativa do processo, facilitando o gerenciamento de resultados e o *benchmarking* entre instituições transplantadoras. O monitoramento contínuo pode otimizar a alocação de recursos e o planejamento de serviços relacionados ao processo de admissão do paciente.

## INTRODUCTION

Liver transplantation began in mid-1963, and specifically in Brazil, in 1968.^(^ ^1^ ^,^ ^2^ ^)^ However, it was only in 1980, with the advancement of surgical techniques, use of immunosuppressors, and prevention of infections that transplants started being indicated routinely for chronic and irreversible hepatic diseases, with the objective of prolonging survival of patients and improving their quality of life.^(^ ^1^ ^–^ ^3^ ^)^

In the past, patients were eligible for liver transplant according to the criteria established by the surgeon. In mid-1997, the National Transplant System (SNT - *Sistema Nacional de Transplante*) and the single waiting list were created, which allowed transparency of the system. As of 2006, the criteria of the Model for End-Stage Liver Disease (MELD) were adopted in Brazil, and their logarithmic calculation is done with laboratory bilirubin tests, international normalized ratio (INR), and creatinine. MELD is a predictor of mortality with the purpose of organizing a list in order of priority, according to severity.^(^ ^4^ ^–^ ^6^ ^)^

The calculation of MELD, the patient's clinical condition, past medical/surgical history, and the complications resulting from the disease, are important aspects that allow evaluation of prognosis, measuring the true benefit of the transplant.^(^ ^2^ ^)^

The admission and enrolment process for the liver transplant list starts with an overall evaluation consisting of laboratory tests, imaging tests, the patient's life history, and psychosocial evaluation, among other specificities. This assessment is made by a specialized multiprofessional team, formed by hepatologist, surgeon, anesthesiologist, nurse, social worker, psychologist, dietitian, and other necessary specialized services. After the meetings, the transplant can be indicated or contraindicated.^(^ ^1^ ^)^ Postoperative survival rate is intimately linked to early referral to this assessment, besides improved surgical techniques, immediate and post-transplant care, and psychosocial follow-up at all times.^(^ ^1^ ^,^ ^7^ ^)^ With the ever-growing demand of patients for liver transplants and the limitation of organ donation, severity of patients on the transplant waiting list increased. This situation also indicates a cost increment in the transplant program due to the complexity of the care delivered, which is related to the pre- or postoperative phases.^(^ ^8^ ^)^

With these changes, the cost-benefit for the programs becomes debatable, and a detailed assessment of efficacy, efficiency and effectiveness of these services is essential, as well as ongoing monitoring of the processes.

In order to maintain the focus on quality, one should continuously seek new sustainable alternatives and assure safety of patients.^(^ ^7^ ^–^ ^9^ ^)^ To evaluate the results of a transplantation program, quality indicators are used. They are statistical values, which point out specific problems that require improvement, aiming to attain goals or perform functions, systems, or processes.^(^ ^9^ ^,^ ^10^ ^)^ They are critical tools focused on the result, always measurable, clear, and objective, and when monitored, they draw attention to deviations of an expected product, allowing visualization of the elements and directing the implementation of measures that impede the possible installation of the problem.^(^ ^9^ ^,^ ^10^ ^)^

Many quality indicators are used, and among the most common are processes that translate the production system.^(^ ^11^ ^)^ Process is the manner in which the resources are used to reach a goal. It is the series of responsible causes for one or more effects perceived as responsible for the final quality of each product.^(^ ^11^ ^,^ ^12^ ^)^ When well planned, systematic, and documented, it transmits safety to follow up each stage, from identification of problems to control of possible solutions. With effective management, it enables evaluating, monitoring, and even modifying processes with the purpose of increasing quality and reducing expenses.^(^ ^13^ ^)^

Another often used tool for reaching good quality is benchmarking, which aims to compare the results of the companies acknowledged for having the best practices, in order to systematically study information to obtain improvements in their organizational environment.^(^ ^14^ ^)^

For a better understanding of the processes related to transplantation, it is possible to adopt a division between the phases (pre-transplant, transplant, and post-transplant), beginning at the first visit to the program, and finalizing with the intercurrent events in the late post-transplant phase.

Within this context, the processes of evaluation and enrolment in the liver transplant program were given priority, and in this phase, systematic analysis, a structured and cohesive team, predefined protocols, and the rigorous control of all steps became crucial. To this end, periodic indicators were used, which could bring palpable results as to the efficiency and efficacy of the actions established, and therefore, able to help in sustainability, quality and safety of the program.^(^ ^1^ ^,^ ^7^ ^,^ ^8^ ^)^

## OBJECTIVE

To establish, measure and analyze quality indicators related to the process of assessment and enrolment of patients who went to a liver transplant program service.

## METHODS

This was a database study with a non-experimental quantitative approach.

The study by De Simone et al., was used as theoretical reference; it was carried out at the Liver Transplant Program of the University of Pisa, Italy, and presents the results reported to the United Network for Organ Sharing (UNOS), demonstrating the flowchart and the mathematical formulas applied, whose results are their quality indicators.^(^ ^7^ ^)^

Data collection was conducted by a nurse of the organization, by means of a non-probabilistic sample, with rational selection and retroactive analysis, extracted from the database of the Liver Transplant Program - *Hospital Israelita Albert Einstein* (HIAE). Information on all patients who sought the outpatient service during the period from September 2012 to September 2014 was gathered, with a total of 1,795 appointments of 1296 patients – the first number includes return visits.

Included in the investigation were all patients seen at outpatient level for assessment of the indication of liver transplantation. No patients were excluded; only return visits.

The demographic aspects, sex, age, and place of origin were also studied, and showed some missing information due to incorrect filling out or lack of data.

**Figure 1:**
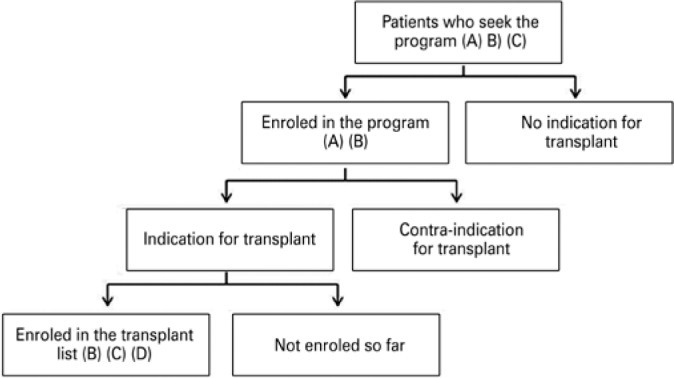
Flowchart of the process of assessment and enrolment of patients for the liver transplant program

The information collected was analyzed by means of a flowchart of the process of admission and evaluation of the potential candidate for a transplant, calculated with simple potentials and with formulas established to measure the indicators. Analysis of the established indicators was extracted from the steps of the process of assessment and enrolment of patients in the liver transplant program, as shown in figure 1.

### Indicator A

#### Patients admitted/total number of patients who sought the program x 100%

For the analysis of efficiency of patient admission in the liver transplant program, we used the rate of patients admitted to the program, after the first assessment of those who sought the service for a more detailed evaluation through laboratory and imaging tests, divided by the total number of those who sought the service for the first time.

### Indicator B

#### Patients enrolled in a list of transplant/admitted patients x 100%

To analyze the efficiency of the assessment of indication for liver transplant, we used the rates of patients enrolled in the technical registry of liver transplants by means of access to data from the São Paulo State Department of Health, divided by the number of patients admitted to the program.

### Indicator C

#### Patients enrolled in a transplant list/total number of patients who sought the program x 100%

For the analysis of efficacy in patient care in the liver transplant program, we used the rates of patients enrolled in the transplant list divided by the total number of those who sought the service.

### Indicator D

#### Time of inclusion in the technical registry as of the first assessment at the service

For the analysis of efficiency in the inclusion of patients in the liver transplant list, the mean and median of the time interval between the first evaluation and inclusion in the waiting list were used.

This investigation was conducted complying with the national and international ethics standards, as per Resolution 466/2012, and was submitted to the Research Ethics Committee of *Hospital Israelita Albert Einstein* and approved by opinion number 888.084, CAAE: 38624814.8.0000.0071. The Informed Consent Form was waived by the committee.

## RESULTS

Among the 1,296 patients who sought the program during the determined period, the mean of return visits was 1.36 per patient, with 1,795 appointments, and 15 (1.2%) of them returned for reevaluation after a certain interval; thus, the sample was made up of 1,311 appointments. Moreover, the sample was composed of 205 (15.6%) patients during the period from September to December 2012, 551 (42%) patients during the period from January to December 2013, and 555 (42.3%) patients during the period from January to September 2014.

Most of the sample was composed of white (854; 65.1%) men (875; 66.7%), considering the variation due to the individual perception of the attendants who registered them in the system (Table 1). The mean age of these patients was 53 (±12.5) years, with a minimum of 13 years and a maximum of 88 years. Most patients (1,059; 80.8%) resided in the State of São Paulo, 690 (52.6%) in the interior of the State, and 369 (28.1%) in the capital city. The others came from several regions of the country.

**Table 1:** Sociodemographic aspects of patients assessed in the liver transplant program of *Hospital Israelita Albert Einstein*

Regions			n (%)	Racial miscegenation	n (%)
Southeast			1196 (91.2)	White	854 (65.1)
	State of São Paulo		1059 (80.8)	Mulatto	280 (21.4)
		São Paulo City	369 (28.1)	Black	94 (7.2)
		Interior	690 (52.6)	Asian	4 (0.3)
Central Western			58 (4.4)	Indigenous	1 (0.1)
Northeast			32 (2.4)	No characterization	78 (5.9)
South			16 (1.2)		
North			9 (0.7)		
**Sex**				**Age**	
Male			875 (66.7)	Mean 53 (±12.5)	
Female			436 (33.3)		

**Figure 2:**
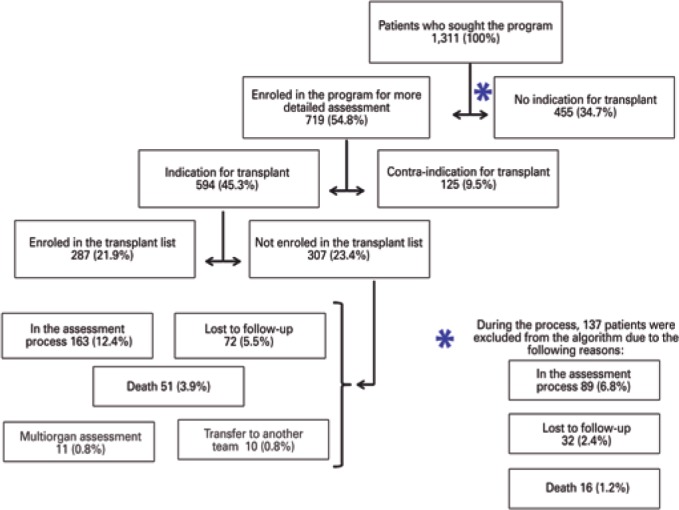
Results of the process of assessment and enrolment of patients for the liver transplant program of *Hospital Israelita Albert Einstein*

As to the indicators, the data was extracted per figure 2.

The results of the quality indicators demonstrated a mean relative to indicator A of 61.2%; relative to indicator B, it was 39.9%; for indicator C, 24.4%; and for indicator D, 107 days (±98 days), with a minimum of one day and a maximum of 633 days, and the median of 84 days (36-142) (Table 2).

**Table 2:** Annual indicators

Indicators	2012 (%)	2013 (%)	2014 (%)
Indicator A	65.3	62.2	43.6
Indicator B	45.5	36.1	42.1
Indicator C	29.7	22.5	18.3
Indicator D (mean/median)	172/144 days	114/87 days	59/43 days

Indicator A: efficiency of patient admission in the liver transplant program; Indicator B: efficiency of the assessment of indication for liver transplant; Indicator C: efficacy in patient care; Indicator D: efficiency in the inclusion of patients in the liver transplant list.

## DISCUSSION

In 2007, a study published on the Quality Progress^(^ ^14^ ^)^ website, highlighted the importance of comparing the results with the excellence services for a better performance of the institutions. The use of benchmarking among the cutting-edge companies and even industries is cited with the intention of using the expertise of other branches to plan new strategies and improve the quality and the safety of the processes, whether relate to care delivery or to other categories.

Bringing this concept to liver transplantation, more specifically the process of admission and evaluation, it was possible to analyze a few indicators. The rate of efficiency of admission in the liver transplantation program, represented by indicator A, was 61.2%, while De Simone et al.,^(^ ^7^ ^)^ found a rate of 44.7% analyzing 1,837 cases. This discrepancy in values can be explained by the volume of patients who arrive at our service without a prior assessment, since in countries such as Italy and the United States, the patient comes for the first assessment in the program only after having been referred by a physician from primary care, that is, already having undergone a brief assessment of their condition, with possible solutions and treatments for their disease already excluded, which enables the evaluation and admission of the patient.^(^ ^1^ ^)^

When compared to the rate of efficiency of the evaluation for referral to liver transplantation, the B indicator, our best result was 39.9% and the international result of 58.8%, resulting from the more specific evaluation mentioned earlier and the eligibility criteria adopted by each country, leading to patients admitted to the international program having been selected in a more well-founded manner, and making the enrolment for a transplant more assertive.^(^ ^7^ ^)^

In reference to indicator C, the efficacy of the patients seen in the program, the results were similar to those found in literature. This parallel may be related to similarities in the active patient monitoring systems among the institutions, decreasing the losses in follow-up and consequently, a greater number of patients enrolled.^(^ ^7^ ^)^

As to the mean time of inclusion in the technical registry - Indicator D - was clearly observed in evolution over the years, resulting in one third of the time that it occurred in 2012, which demonstrates that the efficiency of the process of enrolment in the program has been increasingly improved. Due to the scarcity of literature, it was not possible to establish other comparisons, but as per the regulations established by the Joint Commission on Accreditation of Healthcare Organization*s*, which determines an estimated time of 30 days for the evaluation and forwarding of the candidate to liver transplant, the result of this study still remains above what is expected, with 59 days.^(^ ^15^ ^)^

Several studies were found about quality in transplant, but all focused on the process of the transplant as a whole or specifically to the surgical portion.^(^ ^15^ ^–^ ^18^ ^)^ Also found were various studies on the transplant criteria and mortality in the list. Nevertheless, only one international article was found evaluating quality in the process of patient admission.^(^ ^7^ ^)^

The study performed in the United States and applied to 444 professionals of the active liver transplant programs in the country, emphasized the importance of high level scientific evidence, so that the healthcare services might access and evaluate their results. In this study, it was revealed that the majority of the participating transplantation centers did not have institutional criteria of eligibility for liver transplants. By applying a questionnaire as to knowledge about the eligibility criteria, those who did not have protocols obtained the highest error index, and when questioned, spoke about not having where to search in literature.^(^ ^19^ ^)^

Regarding the demographic data in São Paulo, most patients had come from the interior of the state, since almost 80% of transplantation centers are located in the capital city. The Central Western region was the second one with greatest demand, which may be related to the scarcity of services in this area. The Northeast Region, despite having active transplant programs, still has patients who seek this region perhaps due to the great demographic density, which is counterpoised to the capacity of the transplantation centers. Whereas the Southern Region has large specialized services and presented a low demand; and the North, despite not having many centers, faces logistic difficulties for patients coming to the State of São Paulo. These results are interconnected with deficiency of the transplantation center networks of in certain states of the country.^(^ ^20^ ^)^

Analysis of the indicators allowed a more critical view of the process, demonstrating that some factors such as unavailable agenda for appointments and tests, need for interconsultations and lack of patient orientation, have a direct impact on the agility of the process and become points for ongoing improvements.

The use of appropriate tools should be a continuous action, serving as a strategy for improving the efficiency of the process of admission and the evaluation of the patients, such as ongoing monitoring and logistic planning of the team and resources.

Opening new transplantation centers distributed regionally throughout the country would disseminate the demand of the patients who need a transplant, allowing the access of all, with equity, equality, and universality.

This study presented a few methodological limitations due to the differences in the admission processes at each transplantation center, hindering the standardization of the quality indicators. In literature, we did not find Brazilian studies with similar processes for comparisons of the results. It also was not possible to compare the 2014 indicators due to the period studied.

## CONCLUSION

The quality indicators analyzed in this study will enable a quantitative view of the process, facilitating the comparison between results and benchmarking among the transplant institutions. Ongoing monitoring was able to optimize the allocation of resources and the planning of the services related to the patient admission process.
